# Editorial: Bridging the gap: an interdisciplinary perspective on ketamine in psychiatric disorders

**DOI:** 10.3389/fpsyt.2023.1246891

**Published:** 2023-08-14

**Authors:** Glenn Hartelius, Sherry-Anne Muscat, Lucie Bartova

**Affiliations:** ^1^Attention Strategies Institute, Berkeley, CA, United States; ^2^Youth Forensic Psychiatry, Alberta Hospital, Edmonton, AB, Canada; ^3^Department of Psychiatry and Psychotherapy, Medical University of Vienna, Vienna, Austria

**Keywords:** ketamine for depression, ketamine-assisted psychotherapy, intranasal esketamine, treatment-resistant depression (TRD), antidepressant therapy, brexpiprazole, hypertension management, ketamine psychedelic protocol

While ketamine has emerged as a promising therapeutic for treatment-resistant depression (TRD) and psychiatric emergencies, including suicidality occurring in the course of major depressive disorder (MDD), several issues pertaining to clinical efficacy remain not fully elucidated. These issues include (1) the role of ketamine-assisted psychotherapy (KAP) in enhancing or prolonging antidepressant effects; (2) the therapeutic value of the so-called psychedelic protocols that value the state-altering properties of ketamine; (3) the therapeutic potential of ketamine as a first-line treatment for MDD, other mood diseases, and potentially other psychiatric diagnoses; and (4) the safety and efficacy of home treatment with self-administered ketamine.

This collection of articles extends the currently limited evidence for the efficacy of KAP for MDD, the impact of protocols using doses that induce psychedelic experiences, and the benefits of using both of these approaches with a range of psychiatric diagnoses. The literature on two relatively new treatment options is enriched by a case report illustrating the efficacy of intranasal esketamine, and a study providing early data on safety and efficacy with home use of self-administered oral ketamine. Two frequently discussed risks associated with ketamine treatment are presented with a real-world study examining ketamine's potential for abuse and one study providing guidance for the management of hypertension that may occur in the course of antidepressant treatment with intravenous ketamine. A final case report documented the administration of intranasal esketamine in a 17-year-old female adolescent suffering from TRD, and two articles examined the evidence for differences in the antianhedonic response to ketamine between male and female participants and between individuals with melancholic and non-melancholic depression, representing clinically relevant conditions that are frequently described as predominantly biologically determined manifestations of MDD (1, 2).

In contrast with the scores of studies demonstrating ketamine's efficacy as a fast-acting antidepressant, there is relatively little research on ketamine administered in combination with psychotherapy. There is clear evidence that the addition of psychotherapy to conventional antidepressant psychopharmacotherapy enhances benefits in MDD patients (3–5), although it does not consistently lead to improved outcomes, especially in patients who are more severely ill (6). Given that ketamine treatment shows efficacy with a substantive percentage of TRD patients (7–11), an added psychotherapy component may prove to be highly effective in selected patient populations.

To date, the most robust evidence for the efficacy of KAP using a psychedelic protocol applied to a wide range of mood and trauma-related conditions comes from a retrospective study of 235 adult patients in three separate private general psychology practices in Northern California and Texas (12). The most common diagnoses in this varied group were MDD and complex post-traumatic stress disorder (cPTSD), and the treatment varied from 1 to 25 sessions. Ketamine was typically administered in doses of either 200–250 mg via sublingual injection or 80–90 mg via intramuscular injection. Mean depression scores on the Beck Depression Inventory (BDI) and Hamilton Anxiety Scale (HAM-A) decreased by more than 50% across this population, with the largest improvements in cases involving cPTSD or developmental trauma. Adverse effects of nausea, vomiting, and agitation were reported by a small percentage of patients, only rarely resulting in the discontinuation of treatment. This study highlights the therapeutic value of pairing psychotherapy with ketamine treatment and points to the real-world potential of KAP at psychedelic doses as a treatment for individuals with a variety of diagnoses.

In a different approach, an overlay of a 10-week, 12-session course of cognitive behavioral therapy (CBT) on a short 2-week course of four ketamine infusions (0.5 mg/kg over 40 min) in an open-label trial resulted in remission in 7 out of 16 self-selected patients with TRD within the first 2 weeks (13); for ketamine responders (8/16), average time to relapse was 12 weeks post-ketamine treatment. This differs from less durable results of ketamine-only treatments in a review of nine trials with patients with TRD using ketamine infusions of varying dosages and frequency; in these trials, the mean time to relapse after ketamine treatment ranged from 16 days to 24 days (14). Wilkinson et al. (13) interpreted their evidence as favorable to the use of psychotherapy as a relapse prevention strategy.

In a novel automated psychosocial intervention (15), 154 adult patients with moderate-to-severe treatment-resistant MDD and below-normal self-reported self-esteem received a single ketamine infusion (0.5 mg/kg over 40 min) followed by eight computer-based sessions of a conditioning intervention designed to reinforce an association between positive traits and the patient's self-perception, both supraliminally and subliminally (automated self-association training; ASAT). Interventions took place in a research office setting, with each intervention lasting 15–20 min and occurring twice on four consecutive days with a 20-min interval between sessions. MADRS depression scores remained stable at a low level over the 30 days of the intervention, with the end-of-study effect size favoring the ketamine + ASAT training against the saline infusion + ASAT training and the ketamine infusion + sham training by a small effect size.

New preliminary evidence for KAP with adolescents is provided in “*Ketamine-assisted psychotherapy in adolescents with multiple psychiatric diagnoses*” by Wolfson et al.. Their study reports on four cases of adolescents aged 13–19 years with conditions ranging from TRD, anxiety, and bipolar disorders to trauma and eating disorders; all adolescents were treated with sublingual ketamine followed by sessions with intramuscular ketamine, at individualized and incrementally higher doses until an experience of ego dissolution was achieved, leading to rapid functional improvements and decreases in symptoms.

The benefits of KAP for the treatment of multiple psychiatric diagnoses are further illustrated, along with an insight into the subjective experience of psychedelic-dose ketamine, in “*Medical student types journals during ketamine infusions for suicidal ideation, treatment-resistant depression, post-traumatic stress disorder, and generalized anxiety disorder*” by Willms et al.. In their study, a 30-year-old man had suffered from suicidal ideation for 5 years, despite prior psychotherapy, lifestyle modifications, and complex psychopharmacology. After an 8-month regimen of ketamine infusions at 1.8 to 2.1 mg/kg/h for 1 h, he experienced remission from suicidality, PTSD, MDD, and GAD. The case includes detailed journals of his subjective experience during four 1-h infusions. While evidence for the relationship between ketamine's alterations of experience and its therapeutic efficacy is mixed (16–19), this study adds rare subjective reports of the client experience.

Ketamine treatment frequently provides sustained clinical improvements independent of psychotherapy, as illustrated by “*Intranasal esketamine for severe major depressive disorder with psychotic features*” by Carter et al.. In their study, a 29-year-old patient with TRD, anhedonia, auditory hallucinations, and suicidal thoughts received 14 intranasal esketamine treatments over 3 months. Her depression symptoms were reduced from severe to mild, and her suicidal ideation and auditory hallucinations resolved; moreover, she continued to be stable for 1 year after treatment.

Many of the efforts to improve ketamine's efficacy are aimed at adjusting treatment protocols or combining ketamine treatment with psychotherapy. A different line of innovation combines ketamine with other drugs, as reported in “*Effectiveness of brexpiprazole and esketamine/ketamine combination: a novel therapeutic strategy in five cases of treatment-resistant depression*” by Chan et al.. Brexpiprazole represents a second-generation antipsychotic agent that, similar to ketamine, can be effectively employed in the course of add-on or augmentation treatment in MDD and that impacts the glutamatergic system with the rapid onset of action. A case series illustrates the potential of synergetic mechanisms offered by this novel drug combination.

A recently pioneered treatment option is sublingual ketamine delivered by mail for self-administration at home. A summary of outcomes with 664 patients who completed at least three ketamine sessions appears in “*Safety, effectiveness and tolerability of sublingual ketamine in depression and anxiety: a retrospective study of off-label, at-home use*” by Hassan et al.. After three twice-weekly treatments with rapid dissolve ketamine tablets delivering 300–450 mg—a range that often induces a psychedelic experience (an altered state that may be accompanied by changes in sense perception and/or sense of self)—the mean depression score for this sample decreased by 47.59% as measured by the Patient Health Questionnaire (PHQ-9), with similar decreases in anxiety (GAD-7). After six treatments, the PHQ-9 scores dropped to scores of half or less on intake in 65.4% of the 210 patients who completed this extended protocol, with comparable reductions in anxiety. Minor side effects and adverse events such as dizziness and nausea were limited and resolved without medical intervention, providing evidence that, for some patients, this treatment option may offer added convenience, increased privacy, and reduced cost along with good efficacy and safety.^1^

With the rise of at-home treatment comes an appropriate interest in the addiction and abuse potentials of ketamine, which in addition to its clinical uses is also a popular party drug. These concerns are addressed in “*A survey of drug liking and cravings in patients using sublingual or intranasal ketamine for treatment resistant depression: a preliminary evaluation of real-world addictive potential*” by Chubbs et al.. A survey of 33 patients with TRD in current or prior treatment with sublingual or intranasal ketamine found that ketamine was not consistently liked or craved by these patients. While non-parenteral uses of ketamine require diligent monitoring by providers, abuse concerns should be weighed against the potential benefits of treatment. These findings are consistent with other research pointing to negligible risks of abuse associated with ketamine treatment of mood disorders in patients with no comorbid substance use disorders (21).

Another risk factor that is frequently discussed in terms of ketamine treatment is the potential for transient hypertensive episodes. One of the advantages of ketamine as an anesthetic is that, unlike most anesthetics, it does not depress respiration; this makes it an ideal anesthetic for battlefields and other contexts where close monitoring of patients may be difficult. Conversely, ketamine's propensity to raise blood pressure creates risks for individuals prone to hypertension; while hypertension is not necessarily a contraindication for ketamine treatment, it is a frequent comorbid condition that requires appropriate antihypertensive treatment (20). “*Intravenous ketamine for depression: a clinical discussion reconsidering best practices in acute hypertension management*” by Yip et al. offers best practice guidelines for the management of hypertension, which in rare cases may result from ketamine infusions and even less frequently with intranasal esketamine treatment (20, 22).

The case report “*Ketamine as therapeutic option in TRD in minors*” by Skala et al. portrays a 17-year-old student treated with intranasal ketamine at the recommended dose of 28 mg designed to minimize psychoactive effects. After multiple sessions, treatment was discontinued early due to insignificant improvement in assessments of functioning and depression. Some percentage of the treated individuals do not respond to ketamine, and including reports of these in the literature is valuable for understanding how such cases present and progress, providing insights for further treatment optimizations.

Finally, an important dimension of ongoing research into the effects of ketamine is the testing of similar protocols on different populations. A series of six intravenous infusions of subanesthetic ketamine showed no important differences in the antianhedonic effect on male vs. female participants or individuals with melancholic vs. non-melancholic depression, in two respective articles by Zheng et al.(a) and Zheng et al.(b): “*Gender differences in the antianhedonic effects of repeated ketamine infusions in patients with depression*” and “*A comparison of the antianhedonic effects of repeated ketamine infusions in melancholic and non-melancholic depression*”.

To date, much of the research on ketamine has focused on the efficacy of one or more series of ketamine only treatments for depression, with doses designed to minimize alterations of mental state. These Articles represent research that engages with a number of additional promising dimensions of ketamine therapy. We offer these in hopes that this will spur further interest, richer dialogue, and a wider range of meticulous studies.

## Author contributions

GH conceptualized and wrote the first draft of the manuscript. S-AM and LB contributed to the manuscript revision and read and approved the submitted version. All authors contributed to the article and approved the submitted version.

## Footnotes

^1^This treatment approach is in tension with recommendations that suggest close medical monitoring by psychiatrists responsible for confirming indications for the ketamine antidepressant treatment to avoid potentially inappropriate or not indicated administrations (20).
